# Botanical and geographical origin of Turkish honeys by selected‐ion flow‐tube mass spectrometry and chemometrics

**DOI:** 10.1002/jsfa.10244

**Published:** 2020-01-18

**Authors:** Gulsah Ozcan‐Sinir, Omer U Copur, Sheryl A Barringer

**Affiliations:** ^1^ Faculty of Agriculture, Department of Food Engineering Bursa Uludag University Bursa Turkey; ^2^ Department of Food Science and Technology The Ohio State University Columbus OH USA

**Keywords:** SIFT‐MS, honey aroma, chestnut, heather, wildflower

## Abstract

**BACKGROUND:**

Honey has a very important commercial value for producers as a natural product. Honey aroma is formed from the contributions of several volatile compounds, which are influenced by nectar composition, botanical origins, and location. Selected‐ion flow‐tube mass spectrometry (SIFT‐MS) is a technique that quantifies volatile organic compounds simply and rapidly, even in low concentrations. In this study, the headspace concentration of eight monofloral (chestnut, rhododendron, lavender, sage, carob, heather, citrus, and pine) and three multiflower Turkish honeys were analyzed using SIFT‐MS. Soft independent modeling of class analogy (SIMCA) was used to differentiate honey samples based on their volatiles.

**RESULTS:**

This study focused on 78 volatile compounds, which were selected from previous studies of selected honeys. Very clear distinctions were observed between all honeys. Interclass distances greater than 8 indicate that honeys were significantly different. Methanol and ethanol were abundant in the honeys. Chestnut honey collected from the Yalova region had the highest total concentration of volatiles followed by heather honey and chestnut honey collected from the Düzce region.

**CONCLUSION:**

Honeys with different botanical and geographical origins showed differences in their volatile profile based on chemometric analysis. Of the honey samples, methanol, ethanol, acetoin, ethyl acetate, and isobutanoic acid had the highest discriminating power. Methanol and ethanol, and then acetic acid, were the volatiles with the highest concentrations in most honeys. © 2020 The Authors. *Journal of The Science of Food and Agriculture* published by John Wiley & Sons Ltd on behalf of Society of Chemical Industry.

## INTRODUCTION

Honey is one of the oldest foods in existence, and is consumed not only for its effects on health but also for its taste, nutritional value, and unique flavor. Honey contains mainly water and sugar.^1^ Vitamins, minerals, enzymes, free amino acids, and plentiful volatile compounds are present as secondary constituents.^1^ Different honeys have different minor compound compositions due to their botanical and geographical origin, harvesting season, and processing conditions. The variation in this composition can be used to identify botanical and geographical origins as well as their quality.^2^

Honey has a very important commercial value for producers as a natural product.^3^ World honey production was 1.786.996 t in 2016 and Turkey ranks second after China with 105.532 t.^4^ The most widely found honey types in Turkey are wildflower, pine, chestnut, thyme, linden, citrus, cotton, and sunflower honey.^5^

Volatile compounds present in honey are characteristic markers of botanical origin.^6^, ^7^ Various volatile compounds and representative chemical groups are present at high levels in different honeys.^7^ Some of the main marker compounds are 3‐hydroxy‐5‐methyl‐2‐hexanone, methyl anthranilate, and sinensal isomers in citrus honey, nerolidol oxide, coumarin, hotrienol, hexanal, and hexanol in lavender honey, and 2‐cyclopenten‐1,4‐dione, 2‐aminoacetophenone, 2‐hydroxyacetophenone, guaiacol, propyl anisol, *p*‐anisaldehyde, and *p*‐cresol in heather honey.^8^ Chestnut honeys are noticeable for their high concentrations of acetophenone, 1‐phenylethanol, and 2‐aminoacetophenone,^7^ while lilac aldehyde and 2‐aminoacetophenone are indicators for rhododendron.^9^ According to Tananaki *et al*.,^10^ octanal, 3‐carene, camphene, octane, nonanal, decanal, *α*‐pinene, *β*‐pinene, toluene and 1.2.3‐trimethylindene are marker compounds for pine honey. Characteristic volatiles of sage honey were tetrahydro‐2,2,5,5‐tetramethylfuran, lilac aldehyde, 2‐methylbenzene, heptanoic acid, and benzeneacetic acid.^11^

The evaluation of the botanical and geographical origin of honey is very complicated. The fingerprint of specific honey samples can be determined by measuring organoleptic properties, melissopalynological characteristics, and physicochemical characteristics.^12^ Alternative and faster methods are being considered for characterization of non‐volatile and volatile markers of unifloral honeys.^6^ Unifloral honey aroma is mainly formed by a nectar of the specific flower. Selected‐ion flow‐tube mass spectrometry (SIFT‐MS) is a fast and sensitive analytical technique for real‐time analysis of trace gases by using the chemical ionization of the target gases.^13^

The principal objective of this study was to determine if chestnut, rhododendron, lavender, sage, carob, heather, citrus, pine, and wildflower honeys can be distinguished based on their volatile organic compounds using a SIFT‐MS technique combined with multivariate statistical analysis.

## MATERIALS AND METHODS

### Honey samples and their botanical origin

Twelve honey samples were selected from different parts of Turkey (Table 1). Local beekeepers collected the honey from bees that were kept in hives near fields containing predominantly chestnut, rhododendron, lavender, sage, carob, heather, citrus, pine, or mixed wildflower (Table 1).

**Table 1:** Botanical and geographical origin of honey samples

	Botanical origin	Botanical name	Geographical origin (Turkey)	Abbreviations
1	Chestnut/monofloral	*Castanea sativa* Mill.	Yalova	CY
2	Chestnut/monofloral	*Castanea sativa* Mill.	Düzce	CD
3	Rhododendron/monofloral	*Rhododendron ponticum* L.	Düzce	RD
4	Lavender/monofloral	*Lavandula stoechas* L.	Burdur	LB
5	Sage/monofloral	*Salvia officinalis* L.	Burdur	SB
6	Carob/monofloral	*Ceratonia silique* L.	Antalya	CaA
7	Heather/monofloral	*Calluna vulgaris* L.	Antalya, Alanya	HA
8	Citrus/monofloral	*Citrus* Spp.	Antalya, Kumluca	CiA
9	Pine/monofloral	*Pinus brutia* L.	Muğla, Köyceğiz	PM
10	Wildflower/multifloral	*	Ardahan	WA
11	Wildflower/multifloral	**	Sivas	WS
12	Wildflower/multifloral	***	Kırşehir	WK

* Mixture of *Fraxinus excelsior* L., *Acer platanoides* L., *Cirsium arvense* L., *Cotoneaster sp*., *Fraxinus excelsior* L., *Hedysarum varium*, *Lonicera caucasica*, *Marrubium astracanicum*, *Medicago sativa* L., *Phlomis pungens*, *Prunus spinosa L. subsp* . *dasyphylla*, *Rosa canina* L., *Rubus idaeus* L., *Satureja hortensis* L., *Tilia rubra* DC subsp. *caucasica*, *Vicia sativa* L.

** Mixture of *Anthemis tinctoria* L., *Astragalus* L., *Carduus nutans* L., *Centaurea solstitialis* L., *Centaurea triumfettii*, *Cirsium arvense* L., *Cotoneaster sp*., *Crataegus tanacetifolia* , *Crataegus orientalis* , Eleagnus angustifolia L., *Lonicera caucasica*, *Marrubium astracanicum*, *Morus alba* L., *Onobrychis tournefortii*, *Origanum vulgare* L., *Quercus robur* L., *Rosa canina* L., *Rubus canescens* DC, *Satureja hortensis* L.

*** Mixture of *Acer campestre* L., *Anthemis tinctoria* L., *Carduus nutans* L., *Cistus sp*., *Cotoneaster sp*., *Euphorbia macroclada*, *Genista sessilifolia*, *Lamium amplexicaule* L., *Lonicera etrusca*, *Phlomis armeniaca*, *Rosa canina* L., *Rubus canescens* DC, *Satureja hortensis* L., *Xeranthemum annuum* L.

### Measurement of volatile concentrations

For each honey sample, 10.02 ± 0.2 g was transferred into a 500 mL Pyrex bottle and capped with an open‐top cap, lined with a polytetrafluoroethylene (PTFE)‐faced silicone septa. The samples were kept in a temperature‐controlled water bath (Precision, Jouan Inc., Winchester, VA, USA) at 50 °C for 60 min to allow equilibration of the volatiles, which were released from the honey samples into the headspace. Samples were measured in triplicate.

A selected‐ion flow‐tube mass spectrometer (SIFT‐MS, V200 Syft Technologies, Christchurch, New Zealand) was used to measure and quantify the volatile compounds in the headspace. Selected‐ion flow‐tube mass spectrometry uses chemical ionization with selected positive reagent ions, H_3_O^+^, NO^+^, and O_2_^+^. The concentration of the volatiles was measured by employing the predetermined reaction rate constant for the volatile with a selected precursor ion and accounting for the dilution of the sample gas into the carrier gas (helium) in the flow tube.^14^ Trace volatile analyte compounds were introduced in the reactor at an optimized sample inlet flow rate of 0.35 Torr·L/s (26 cm^3^ min^−1^).

The range of the mass‐to‐charge ratio was set to 10–250 m/z, with a total SIM scan time of 120 s. The concentration of measured volatile compounds, which was calculated through known kinetic parameters, is listed in Table 2. Concentrations were measured in μg L^−1^ in the headspace above the honey sample. During the analysis, some compounds produce the same mass for a given precursor ion, so the interfering compounds have to be reported as a mixture. In this study, several mixtures were identified at different charge‐to‐mass ratios, such as 2‐methyl‐2‐butanol and butanoic acid at 71 m/z, acetone and isoamyl alcohol at 88 m/z, acetic acid and 2‐cyclopenten‐1,4‐dione at 90 m/z, dimethyl disulfide and phenol at 94 m/z, acetic acid and *p*‐cresol at 108 m/z, acetoin and ethyl acetate at 118 m/z, phenylacetaldehyde and isopropyl benzene at 120 m/z, 2‐phenylethanol and santene at 122 m/z, dimethyl trisulfide and hydroxymethylfurfural at 126 m/z, *α*‐pinene, *β*‐pinene, 2‐hydroxyacetophenone and 4‐methoxybenzaldehyde at 136 m/z, octanoic acid and nonanol at 144 m/z, and hotrienol and *p*‐menth‐1‐en‐9‐al at 152 m/z.

**Table 2:** Kinetics parameters for SIFT‐MS analysis of selected volatile compounds in Turkish honeys

	Compounds	Precursor ion	Product ion	k (10^−9^ cm^3^ s^−1^)	*m/z*
1	(*E*)‐2‐hexenal	NO^+^	C_6_H_9_O^+^	3.8	97
2	(*E*)‐2‐methyl‐2‐butenal	NO^+^	C_5_H_7_O^+^	4.0	83
3	(*Z*)‐3‐hexen‐1‐ol	NO^+^	C_6_H_10_^+^	2.5	82
4	1,3‐butanediol	O_2_^+^	C_4_H_8_O^+^	3.3	72
5	1‐hexanol	NO^+^	C_6_H_13_O^+^	2.4	101
6	1‐octen‐3‐ol	H_3_O^+^	C_8_H_15_^+^	3.1	111
7	1‐*p*‐menthen‐9‐ol	NO^+^	C_10_H_18_O^+^	2.5	154
8	2,3‐butanedione	NO^+^	C_4_H_6_O_2_^+^	1.3	86
9	2‐aminoacetophenone	NO^+^	C_8_H_9_NO^+^	2.4	135
10	2‐butanol	O_2_^+^	C_3_H_6_^+^	2.1	42
11	2‐cyclopenten‐1,4‐dione	NO^+^	C_5_H_4_O_2_^+^	2.5	90
12	2‐heptanol	NO^+^	C_7_H_14_O.NO^+^	3.4	144
13	2‐hydroxyacetophenone	NO^+^	C_8_H_8_O_2_^+^	2.5	136
14	2‐methyl‐2‐butanol	H_3_O^+^	C_5_H_11_^+^	2.8	71
15	2‐phenylethanol	NO^+^	C_8_H_10_O^+^	2.3	122
16	3‐methylbutanal	NO^+^	C_5_H_9_O^+^	3.0	85
17	4‐methoxybenzaldehyde	NO^+^	C_8_H_8_O_2_^+^	2.8	136
18	5‐methylfurfural	NO^+^	C_6_H_6_O_2_^+^	3.1	110
19	acetic acid	NO^+^	NO^+^.CH_3_COOH, NO^+^.CH_3_COOH.H_2_O	0.9	90, 108
20	acetoin	NO^+^	C_4_H_8_O_2_.NO^+^	2.5	118
21	acetone	NO^+^	C_3_H_6_O^+^	1.2	88
22	alpha‐pinene	NO^+^	C_10_H_16_^+^	2.3	136
23	benzaldehyde	NO^+^	C_7_H_5_O^+^	2.8	105
24	benzyl alcohol	NO^+^	C_7_H_7_O^+^	2.3	107
25	beta‐pinene	NO^+^	C_10_H_16_^+^	2.1	136
26	butanoic acid	NO^+^	C_3_H_7_CO^+^	1.9	71
27	chloroform	O_2_^+^	CH(Cl_35_)(Cl_37_) ^+^	1.8	85
28	*cis*‐6‐nonen‐1‐ol	NO^+^	C_9_H_18_O^+^	2.5	142
29	coumarin	O_2_^+^	C_9_H_6_O_2_^+^, C_9_H_6_O_2_.H^+^	2.5	146, 147
30	damascenone	NO^+^	C_3_H_18_O^+^	2.5	190
31	decanal	NO^+^	C_10_H_19_O^+^	3.3	155
32	dimethyl disulfide	H_3_O^+^	(CH_3_)2S_2_.H^+^	2.6	95
33	dimethyl sulfide	O_2_^+^	(CH_3_)_2_S^+^	2.2	62
34	dimethyl trisulfide	O_2_^+^	C_2_H_6_S_3_^+^	2.2	126
35	dodecane	NO^+^	C_12_H_25_^+^	1.5	169
36	ethanol	NO^+^	C_2_H_5_O^+^, C_2_H_5_O^+^.H_2_O, C_2_H_5_O^+^.2H_2_	1.2	45, 63, 81
37	ethyl acetate	O_2_^+^	C_2_H_5_O_2_^+^	2.4	61
38	ethyl benzoate	H_3_O^+^	C_6_H_5_COOC_2_H_5_.H^+^, C_6_H_5_COOC_2_H_5_.H^+^.H_2_O	3.1	151, 169
39	furfural	NO^+^	C_5_H_4_O_2_^+^	3.2	96
40	furfuryl alcohol	NO^+^	C_5_H_6_O_2_^+^	2.5	98
41	guaiacol	NO^+^	C_7_H_8_O_2_^+^	2.5	124
42	heptanal	NO^+^	C_7_H_13_O^+^	3.3	113
43	heptane	H_3_O^+^	C_7_H_16_^+^	0.26	119
44	heptanoic acid	NO^+^	C_7_H_14_O_2_+	2.5	130
45	hexanal	NO^+^	C_6_H_11_O^+^	2.5	99
46	hexane	O_2_^+^	C_6_H_14_^+^	1.76	86
47	hexanoic acid	H_3_O^+^	C_6_H_12_O_2_.H^+^	3.0	117
48	hotrienol	NO^+^	C_10_H_16_O^+^	2.6	152
49	hydroxymethylfurfural	O_2_^+^	C_6_H_6_O_3_^+^, C_6_H_6_O_3_.H^+^	2.5	126, 127
50	isoamyl alcohol	NO^+^	C_5_H_12_O^+^	2.5	88
51	isobutyl alcohol	NO^+^	C_4_H_9_O^+^	2.4	73
52	isopropyl benzene	NO^+^	C_9_H_12_^+^	1.2	120
53	lemonol	NO^+^	C_10_H_17_^+^	2.5	137
54	lilac alcohol	NO^+^	C_10_H_18_O_2_^+^	2.5	170
55	lilac aldehyde	NO^+^	C_10_H_16_O_2_^+^	2.6	168
56	maltol	NO^+^	C_6_H_6_O_3_.NO^+^	2.5	156
57	menthol	NO^+^	C_10_H_19_^+^, C_10_H_19_^+^.2H_2_O	2.6	139, 175
58	methanol	H_3_O^+^	CH_5_O^+^, CH_3_OH2^+^.H_2_O, CH_3_OH.H^+^.(H_2_O)_2_	2.7	33, 51, 69
59	methyl anthranilate	NO^+^	C_8_H_9_NO2^+^	2.5	151
60	nerolidol oxide	NO^+^	C_15_H_26_O_2_^+^	2.5	238
61	nerolidol	NO^+^	C_15_H_26_O^+^	3.0	222
62	nonanal	NO^+^	C_10_H_18_	3.2	138
63	nonane	H_3_O^+^	C_9_H_20_.H_3_O^+^	1.3	147
64	nonanol	NO^+^	C_9_H_20_O^+^	2.5	144
65	octanal	NO^+^	C_8_H_15_O^+^	3.0	127
66	octane	O_2_^+^	C_8_H_18_^+^	1.9	114
67	octanoic acid	NO^+^	C_8_H_16_O_2_^+^	2.5	144
68	*p*‐cresol	NO^+^	C_7_H_8_O^+^	2.2	108
69	*p*‐isopropenyl toluene	O_2_^+^	C_10_H_12_^+^	1.8	132
70	*p*‐menth‐1‐en‐9‐al	NO^+^	C_10_H_16_O^+^	2.5	152
71	phenol	NO^+^	C_6_H_6_O^+^	2.0	94
72	phenylacetaldehyde	NO^+^	C_8_H_8_O.NO^+^	2.5	150
73	phytalic acid	NO^+^	C_8_H_6_O_4_^+^	2.5	166
74	propanoic acid	O_2_^+^	C_2_H_5_COOH^+^	2.2	74
75	propyl anisol	NO^+^	C_10_H_14_O^+^	2.5	150
76	santene	NO^+^	C_9_H_14_^+^	2.5	122
77	toluene	NO^+^	C_7_H_8_^+^	1.7	92

### Statistical analysis

The concentrations of volatile compounds were analyzed in triplicate. One‐way analysis of variance (ANOVA) using Tukey's procedure with a 95% confidence interval was performed to determine statistical differences among samples; significance was defined as *P* ≤ 0.05 using SPSS (version 25, SPSS Inc., Chicago, IL, USA). Multivariate statistical analysis was conducted using SIMCA with Pirouette software for Windows Comprehensive Chemometrics Modeling, version 4.0 (Infometrix Inc., Bothell, WA, USA) to identify distributions of volatiles in honey samples.

## RESULTS AND DISCUSSION

### Volatile composition of honeys

**Figure 1:**
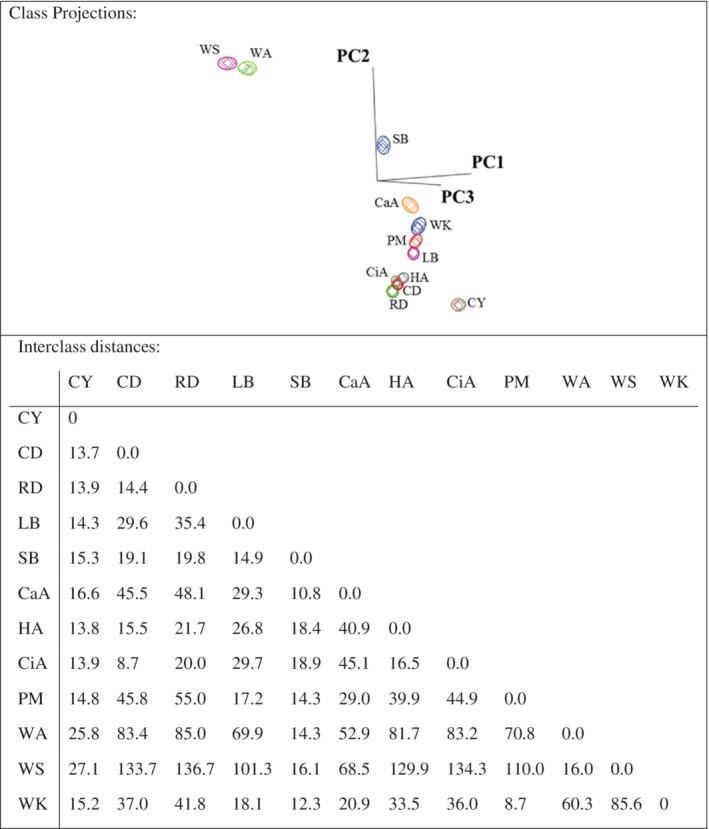
Soft independent modeling of class analogy (SIMCA) 3D projection plots of data collected by SIFT‐MS for Turkish honeys. The SIMCA plots and boundaries marked around the sample clusters represent a 95% confidence interval for each class. Interclass distances between 12 honeys based on the SIMCA class projections.

Many compounds have been detected in honey using different techniques. This study focused on 78 volatile compounds, which were selected from previous studies of selected honeys. Twelve honey samples of known botanical and geographical origins were analyzed (Table 3). Methanol and ethanol, as in other types of honey and food, were abundant in the analyzed samples. Even though these alcohols were commonly found in natural products due to the metabolism of yeasts,^15^ or reduction of aldehydes,^16^ they can be the most effective discriminators based on either their high volatility or discriminating power (Fig. 1). These compounds have been found to discriminate among different type of honeys, such as thyme and lavender honey.^17^

**Table 3:** Concentration (μg L^−1^) of volatile compounds of honeys from different botanical origins and locations

		Chestnut‐Yalova	Chestnut‐Düzce	Rhododendron	Lavender	Sage	Carob	Heather	Citrus	Pine	Wildflower‐Ardahan	Wildflower‐Sivas	Wildflower‐Kırşehir
1	(*E*)‐2‐hexenal	511.23^a^	14.30^b^	9.66^b^	17.44^b^	7.40^b^	10.85^b^	28.53^b^	4.88^b^	7.73^b^	3.43^b^	7.47^b^	3.76^b^
2	(*E*)‐2‐methyl‐2‐butenal	3250.8^a^	240.77^b^	119.24^b^	57.07^b^	17.46^b^	38.32^b^	168.76^b^	30.54^b^	41.92^b^	16.02^b^	13.10^b^	10.10^b^
3	(*Z*)‐3‐hexen‐1‐ol	136.27^a^	49.83^de^	66.38^cd^	79.33^c^	36.49^ef^	101.09^b^	113.04^b^	20.35^fg^	19.27^fg^	8.31^g^	5.45^g^	14.59^g^
4	1,3‐butanediol	757.36^a^	86.57^bc^	53.84^c^	160.15^bc^	101.71^bc^	189.99^b^	133.99^bc^	73.47^bc^	68.51^bc^	61.68^c^	72.94^bc^	47.76^c^
5	1‐hexanol	145.65^a^	20.70^c^	11.30^cd^	57.46^b^	155.71^a^	17.84^cd^	18.85^cd^	8.04^cd^	10.91^cd^	8.82^cd^	11.55^cd^	6.21^d^
6	1‐octen‐3‐ol	79750^a^	441.60^b^	185.30^b^	69.91^b^	35.09^b^	55.66^b^	305.91^b^	75.60^b^	68.28^b^	36.32^b^	40.63^b^	25.94^b^
7	1‐*p*‐menthen‐9‐ol	165.87^b^	5.63^c^	7.60^c^	169.61^b^	352.98^a^	42.39^c^	34.54^c^	7.91^c^	19.42^c^	20.34^c^	32.16^c^	20.59^c^
8	2,3‐butanedione	179.78^b^	87.40^e^	76.94^ef^	109.52^d^	79.78^ef^	208.61^a^	200.44^a^	64.73^fg^	74.56^ef^	129.66^c^	64.20^fg^	51.91^g^
9	2‐aminoacetophenone	2376.6^a^	49.93^b^	14.63^b^	22.48^b^	25.96^b^	11.58^b^	59.36^b^	5.00^b^	6.54^b^	2.29^b^	2.22^b^	1.93^b^
10	2‐butanol	879.22^a^	421.14^c^	168.68^efg^	277.27^de^	189.31^efg^	604.02^b^	604.38^b^	253.44^def^	211.55^defg^	119.97^g^	331.65^cd^	136.03^fg^
11	2‐cyclopente*n*‐1,4‐dione	1058.4^c^	516.50^de^	585.63^d^	444.71^def^	341.16^fg^	1400.9^a^	1217.7^b^	191.93^gh^	387.04^ef^	205.55^gh^	1040.3^c^	179.91^h^
12	2‐heptanol	190.14^a^	15.42^b^	9.39^b^	12.64^b^	14.86^b^	16.98^b^	21.86^b^	6.62^b^	8.59^b^	5.81^b^	6.60^b^	5.38^b^
13	2‐hydroxyacetophenone	286.01^b^	16.65^c^	9.93^c^	578.60^a^	399.65^b^	12.82^c^	53.58^c^	9.73^c^	7.57^c^	3.96^c^	4.74^c^	2.88^c^
14	2‐methyl‐2‐butanol	252.61^c^	79.41^d^	30.81^f^	68.98^de^	35.35^f^	478.26^a^	288.43^b^	54.47^def^	53.69^def^	34.02^f^	70.49^de^	46.64^ef^
15	2‐phenylethanol	3397.9^a^	93.02^b^	37.93^b^	30.06^b^	25.36^b^	39.61^b^	72.50^b^	22.76^b^	29.94^b^	21.46^b^	15.04^b^	14.78^b^
16	3‐methylbutanal	174.47^a^	91.09^c^	25.05^e^	34.00^e^	23.97^e^	183.61^a^	114.35^b^	25.11^e^	69.72^d^	24.99^e^	28.75^e^	18.59^e^
17	4‐methoxybenzaldehyde	283.74^b^	16.51^c^	9.85^c^	574.01^a^	396.48^b^	12.72^c^	53.16^c^	9.65^c^	7.51^c^	3.93^c^	4.70^c^	2.86^c^
18	5‐methylfurfural	109.06^a^	15.05^b^	6.69^b^	20.98^b^	116.66^a^	17.83^b^	18.51^b^	6.71^b^	8.73^b^	5.83^b^	15.84^b^	5.42^b^
19	acetic acid	3707.9^a^	1495.6^cd^	1654.5^c^	1265.6^cde^	988.71^ef^	3916.7^a^	3428.3^a^	542.24^f^	1088.5^de^	579.95^f^	2908.1^b^	505.65^f^
20	acetoin	814.95^b^	230.32^d^	77.41^fg^	158.36^def^	159.49^def^	1655.8^a^	507.46^c^	101.66^efg^	114.08^efg^	39.40^g^	191.30^de^	100.25^efg^
21	acetone	4020.9^a^	1189.0^c^	133.05^e^	2063.7^b^	424.26^de^	471.51^de^	318.68^e^	266.98^e^	950.71^c^	226.45^e^	822.88^cd^	327.30^e^
22	alpha‐pinene	403.75^b^	23.50^c^	14.02^c^	816.77^a^	564.16^b^	18.09^c^	75.64^c^	13.73^c^	10.69^c^	5.60^c^	6.69^c^	4.06^c^
23	benzaldehyde	5720.1^a^	292.29^b^	159.32^b^	72.16^b^	39.47^b^	241.64^b^	455.33^b^	46.88^b^	99.70^b^	17.05^b^	53.24^b^	24.92^b^
24	benzyl alcohol	14383^a^	407.02^b^	215.02^b^	49.97^b^	19.03^b^	122.45^b^	726.06^b^	42.66^b^	64.02^b^	12.66^b^	22.43^b^	15.94^b^
25	beta‐pinene	382.58^b^	22.27^c^	13.28^c^	773.94^a^	534.58^b^	17.14^c^	71.68^c^	13.01^c^	10.13^c^	5.30^c^	6.34^c^	3.85^c^
26	utanoic acid	611.57c	192.25d	74.60f	167.01de	85.58f	1157.9a	698.31b	131.88def	129.99def	82.36f	170.65de	112.92ef
		**Chestnut‐Yalova**	**Chestnut‐Düzce**	**Rhododendron**	**Lavender**	**Sage**	**Carob**	**Heather**	**Citrus**	**Pine**	**Wildflower‐Ardahan**	**Wildflower‐Sivas**	**Wildflower‐Kırşehir**
27	chloroform	3646.5^a^	251.17^b^	151.00^b^	237.60^b^	213.32^b^	274.09^b^	272.06^b^	157.04^b^	136.02^b^	118.52^b^	120.44^b^	80.84^b^
28	*cis*‐6‐nonen‐1‐ol	53.70^b^	13.71^d^	11.17^d^	23.98^c^	10.69^d^	73.33^a^	26.35^c^	8.31^d^	10.67^d^	11.37^d^	9.37^d^	13.67^d^
29	coumarin	2050.9^a^	41.39^b^	16.87^b^	19.16^b^	32.13^b^	46.06^b^	44.48^b^	11.69^b^	11.96^b^	8.73b	7.28b	7.92b
30	damascenone	22.23^a^	8.71b^cd^	6.35^cd^	8.01^cd^	8.66^bcd^	13.62^b^	10.96^bc^	5.66^d^	6.42^cd^	7.61 cd	5.77d	5.89d
31	decanal	50.33^a^	5.47^c^	3.80^c^	26.31^b^	39.69^a^	8.62^c^	6.76^c^	3.54^c^	4.03^c^	4.12^c^	4.21^c^	3.71^c^
32	dimethyl disulfide	26833^a^	140.95^b^	48.05^b^	44.12^b^	14.49^b^	45.64^b^	118.12^b^	14.52^b^	12.53^b^	8.86^b^	17.07^b^	5.08^b^
33	dimethyl sulfide	1447.9^a^	169.72^d^	102.26^d^	161.48^d^	135.36^d^	254.61^bcd^	396.17^bc^	469.57^b^	194.09^cd^	109.46^d^	150.54^d^	157.87^d^
34	dimethyl trisulfide	750.75^a^	35.97^b^	32.14^b^	96.89^b^	132.58^b^	58.82^b^	56.54^b^	32.68^b^	31.56^b^	28.57^b^	37.09^b^	27.95^b^
35	dodecane	393.64^b^	36.73^c^	32.67^c^	320.92^b^	23.23^c^	612.25^a^	348.46^b^	43.67^c^	21.75^c^	21.20^c^	12.46^c^	13.23^c^
36	ethanol	255571^a^	72135^b^	54791^c^	24578^def^	5996.2^g^	19492^ef^	66825^bc^	31769^de^	34235^d^	2465.4^g^	4227.8^g^	11994^fg^
37	ethyl acetate	1077.9^b^	304.66^d^	102.39^fg^	209.48^def^	210.97^def^	2190.2^a^	671.24^c^	134.46^efg^	150.91^efg^	52.11^g^	253.05^de^	132.60^efg^
38	ethyl benzoate	8035.1^a^	59.03^b^	75.88^b^	40.28^b^	25.84^b^	44.78^b^	67.80^b^	85.78^b^	29.39^b^	22.02^b^	17.02^b^	12.46^b^
39	furfural	409.49^a^	23.09^d^	18.53^d^	201.01^b^	54.19^cd^	49.41^cd^	65.10^cd^	16.33^d^	32.33^d^	25.32^d^	82.90^c^	23.10^d^
40	furfuryl alcohol	232.70^a^	60.77^b^	17.33^c^	15.82^c^	26.67^c^	24.12^c^	21.98^c^	8.25^c^	17.00^c^	6.59^c^	29.18^bc^	7.45^c^
41	guaiacol	24.55^a^	6.20^de^	5.01^de^	13.01^b^	11.00^bc^	7.73^cd^	5.91^de^	4.14^de^	5.98^de^	5.14^de^	4.83^de^	3.87^e^
42	heptanal	66.38^a^	16.81^c^	11.67^c^	16.52^c^	16.07^c^	34.03^b^	19.91^c^	9.07^c^	13.59^c^	13.08^c^	11.17^c^	14.55^c^
43	heptane	26034^a^	356.61^b^	170.89^b^	250.61^b^	175.48^b^	191.74^b^	506.44^b^	94.52^b^	152.05^b^	80.83^b^	144.31^b^	63.46^b^
44	heptanoic acid	84.37^a^	39.72^b^	27.74^bc^	33.89^bc^	24.58^bc^	32.36^bc^	27.69^bc^	22.08^c^	29.87^bc^	33.79^bc^	33.86^c^	20.52^bc^
45	hexanal	92.95^a^	28.31^e^	20.51^efg^	45.74^d^	78.18^b^	59.64^c^	27.17^ef^	11.62^g^	17.15^fg^	12.18^g^	18.75^efg^	10.35^g^
46	hexane	1523.9^a^	212.59^bcd^	145.33^cd^	285.60^bcd^	166.75^cd^	391.68^bc^	447.35^b^	155.27^cd^	165.56^cd^	185.42^cd^	138.98^d^	116.88^d^
47	hexanoic acid	4595.9^a^	116.13^b^	62.45^b^	82.39^b^	41.91^b^	119.65^b^	128.24^b^	47.55^b^	52.92^b^	44.75^b^	42.51^b^	34.52^b^
48	hotrienol	101.36^bc^	13.75^c^	14.30^c^	193.68^b^	501.89^a^	23.91^c^	17.86^c^	15.81^c^	15.36^c^	9.71^c^	16.00^c^	8.86^c^
49	hydroxymethylfurfural	1694.2^a^	32.68^b^	27.53^b^	82.47^b^	66.06^b^	44.75^b^	51.58^b^	24.31^b^	23.97^b^	19.43^b^	26.05^b^	18.86^b^
50	isoamyl alcohol	1913.9^a^	564.05^c^	63.09^e^	978.32^b^	201.24^de^	223.52^de^	151.38^e^	126.66^e^	450.72^c^	107.33^e^	390.23^cd^	155.16^e^
51	isobutyl alcohol	18581^a^	387.24^b^	170.24^b^	52.87^b^	19.90^b^	103.67^b^	477.65^b^	81.01^b^	78.82^b^	22.29^b^	49.95^b^	30.45^b^
52	isopropyl benzene	440.40^a^	53.87^cd^	33.81^d^	82.20^bcd^	122.60^b^	88.46^bc^	81.83^bcd^	94.00^bc^	45.60^cd^	45.76^cd^	35.50^d^	116.69^b^
53	lemonol	18338^a^	162.01^b^	53.86^b^	493.91^b^	188.40^b^	10.22^b^	139.64^b^	13.95^b^	21.94^b^	16.03^b^	6.23^b^	10.91^b^
		**Chestnut‐Yalova**	**Chestnut‐Düzce**	**Rhododendron**	**Lavender**	**Sage**	**Carob**	**Heather**	**Citrus**	**Pine**	**Wildflower‐Ardahan**	**Wildflower‐Sivas**	**Wildflower‐Kırşehir**
54	lilac alcohol	35.71^b^	9.77^c^	8.37^c^	29.18^b^	8.35^c^	49.88^a^	29.27^b^	7.54^c^	8.52^c^	8.27^c^	6.05^c^	6.62^c^
55	lilac aldehyde	65.37^a^	5.18^f^	7.39^ef^	24.71^c^	6.87^ef^	14.86^de^	35.16^b^	18.85^cd^	4.37^f^	5.79^ef^	7.12^ef^	5.22^f^
56	maltol	62.02^b^	22.43^e^	29.22^de^	86.34^a^	35.92^d^	77.60^a^	48.85^c^	31.76^de^	23.80^e^	23.26^e^	39.48^cd^	21.29^e^
57	menthol	96905^a^	98.89^b^	35.92^b^	55.62^b^	53.41^b^	28.70^b^	95.57^b^	16.23^b^	19.99^b^	13.48^b^	13.13^b^	12.34^b^
58	methanol	13011^a^	5453.16^ef^	2659.7^h^	4812.8^fg^	4539.2^fg^	7437.1^cd^	6325.8^de^	2699.1^h^	8611.5^bc^	4838.0^fg^	8833.9^b^	3828.3^gh^
59	methyl anthranilate	330.50^a^	6.06^b^	6.88^b^	14.92^b^	13.90^b^	7.97^b^	9.23^b^	15.67^b^	3.10^b^	1.34^b^	1.21^b^	1.42^b^
60	nerolidol oxide	5.59^a^	2.19^b^	1.94^b^	1.88^b^	2.49^b^	1.97^b^	1.51^b^	1.41^b^	1.83^b^	1.93^b^	1.77^b^	1.37^b^
61	nerolidol	4.23^a^	1.57^bcd^	1.09c^d^	1.18^cd^	3.11^ab^	2.77^abc^	1.25^bcd^	1.05^cd^	1.11^cd^	1.35^bcd^	0.80^d^	0.94^cd^
62	nonanal	15035^a^	179.67^b^	79.23^b^	303.33^b^	97.16^b^	249.98^b^	325.49^b^	48.67^b^	67.08^b^	35.51^b^	43.67^b^	38.41^b^
63	nonane	7288.7^a^	118.15^b^	58.74^b^	61.42^b^	41.08^b^	99.86^b^	110.54^b^	31.47^b^	42.13^b^	29.21^b^	28.86^b^	23.43^b^
64	nonanol	103.86^a^	23.72^b^	20.34^b^	27.48^b^	18.52^b^	19.45^b^	21.22^b^	15.68^b^	18.76^b^	27.18^b^	17.52^b^	20.44^b^
65	octanal	158.50^a^	12.84^b^	11.50^b^	22.72^b^	14.79^b^	17.84^b^	21.17^b^	11.95^b^	11.80^b^	10.38^b^	17.77^b^	9.08^b^
66	octane	564.11^a^	66.00^b^	48.70^b^	60.37^b^	43.10^b^	59.44^b^	64.67^b^	34.97^b^	47.78^b^	46.66^b^	45.17^b^	38.29^b^
67	octanoic acid	103.86^a^	23.72^b^	20.34^b^	27.48^b^	18.52^b^	19.45^b^	21.22^b^	15.68^b^	18.76^b^	27.18^b^	17.52^b^	20.44^b^
68	*p*‐cresol	314.18^a^	24.90^b^	11.36^b^	12.37^b^	16.79^b^	10.30^b^	18.68^b^	3.72^b^	5.50^b^	3.67^b^	7.45^b^	2.42^b^
69	*p*‐isopropenyl toluene	509.32^a^	49.59^b^	13.40^b^	11.52^b^	6.88^b^	18.51^b^	15.21^b^	6.45^b^	9.62^b^	10.36^b^	6.42^b^	6.28^b^
70	*p*‐menth‐1‐en‐9‐al	105.42^bc^	14.29^c^	14.87^c^	201.43^b^	521.96^a^	24.87^c^	18.58^c^	16.45^c^	15.97^c^	10.10^c^	16.64^c^	9.22^c^
71	phenol	32200^a^	169.14^b^	57.66^b^	52.95^b^	17.38^b^	54.77^b^	141.75^b^	17.43^b^	15.04^b^	10.63^b^	20.49^b^	6.09^b^
72	phenylacetaldehyde	211.39^a^	25.86^cd^	16.23^d^	39.46^bcd^	58.85^b^	42.46^bc^	39.28^bcd^	45.12^bc^	21.89^cd^	21.97^cd^	17.04^d^	56.01^b^
73	phytalic acid	37.09^a^	3.38^c^	2.87^c^	32.63^a^	22.51^b^	5.42^c^	6.80^c^	3.42^c^	2.79^c^	3.55^c^	2.61^c^	2.64^c^
74	propanoic acid	5329.5^a^	207.51^b^	56.07^b^	61.61^b^	43.09^b^	272.19^b^	179.76^b^	41.94^b^	64.48^b^	40.49^b^	64.49^b^	37.38^b^
75	propyl anisol	116.41^a^	9.38^cd^	24.81^b^	15.01^bcd^	16.21^bc^	7.81^cd^	6.48^cd^	8.99^cd^	9.84^cd^	5.63^cd^	3.92^d^	10.31^cd^
76	santene	259.19^a^	30.71^b^	19.90^b^	12.70^b^	12.95^b^	16.44^b^	12.65^b^	13.61^b^	12.42^b^	16.42^b^	9.68^b^	8.05^b^
77	toluene	8161.9^a^	515.01^b^	244.24^bc^	117.79^bc^	49.25^bc^	43.65^bc^	432.70^bc^	86.34^bc^	80.62^bc^	10.18^c^	15.93^c^	23.08^c^

Superscript letters in the row indicate statistically significant differences (*P* < 0.05).

Acetic acid was the third highest concentrated volatile followed by methanol and ethanol in the honeys, except for chestnut honeys from Yalova region and lavender (Table 3). Acetic acid is formed through degradation of alcohols and produce acidic aroma in honey.^18^ Menthol was the second highest compound for chestnut honey from Yalova and phenol was third followed by 1‐octen‐3‐ol. The acetone concentration was the third highest in lavender honey. Menthol is a mint essential oil, which is allowed to be used in formulations against mites and ticks.^19^

Acetone was the fourth highest volatile of chestnut_Düzce, pine, wildflower_Ardahan and wildflower_Kırşehir, while 2‐cyclopenten‐1,4‐dione was the fourth leading volatile compound for rhododendron, heather and wildflower_Sivas. Alpha‐pinene was the fourth higest concentrated compound measured in sage honey, while ethyl acetate was measured in carob and dimethyl sulfide in citrus as fourth highest compound. Acetone is responsible for a pungent or fruity odor and ethyl acetate gives a fruity aroma in honey.^20^ In the presence of ethanol, ethyl acetate is formed through esterification of acetic acid via microorganisms.^21^ Alpha‐pinene was one of the compounds detected in the honey profile that comes directly from the flower.^22^

Ethyl acetate, which is the ester formed from ethanol and acetic acid, was one of the most abundant compounds in carob honey after ethanol, methanol, and acetic acid. Some studies have focused on the volatile compounds found in carob;^23^, ^24^ however, only one published article focused on the volatile characteristics of carob honey, which is mainly characterized by nonanal and octanal.^25^ Heather, citrus, wildflower honey from Sivas, and wildflower honey from Kırşehir also had high amounts of ethyl acetate.

Dimethyl sulfide was one of the compounds with the highest concentration detected in citrus after ethanol, methanol, and acetic acid. It was also found in both raw and heat‐treated citrus honey from Spain.^26^ The concentration of dimethyl sulfide was relatively high in rare unifloral honeys in Spain such as *Persea americana* (38.5%), *Spartocytisus supranubius* (25.2%), *Quercus ilex* (7.4–337%), *Satureja montana* (22.8%), and *Agave* honey (19.4%).^27^

Hotrienol is one of the marker volatiles for lavender honey;^8^, ^28^ however, in our study it was of a lower concentration compared to other compounds. Sage honey had a significantly higher amount of hotrienol than other honeys (Table 3); however, previous studies did not report it in sage honey.^11^, ^29^ The geographic areas of the lavender and sage honey were in the same province, which may lead the bees harvesting from both area and caused similarities in volatile composition. Hotrienol comes from the flower, during ripening of the honey in the hive and is thermally generated during pasteurization.^30^

Several alcohols were identified in lavender honey, and ethanol, methanol, isoamyl alcohol, lemonol, 2‐butanol, hotrienol and 1,3‐butanediol were the highest concentrations. Radovic *et al*.^31^ determined that ethanol, 2‐methyl‐1‐propanol, 3‐methyl‐1‐butanol, 3‐methyl‐3‐buten‐1‐ol, hotrienol, and furfuryl alcohol were the main alcohols present in lavender honey collected from France and Portugal.

### Effect of botanical and geographical origins

**Figure 2:**
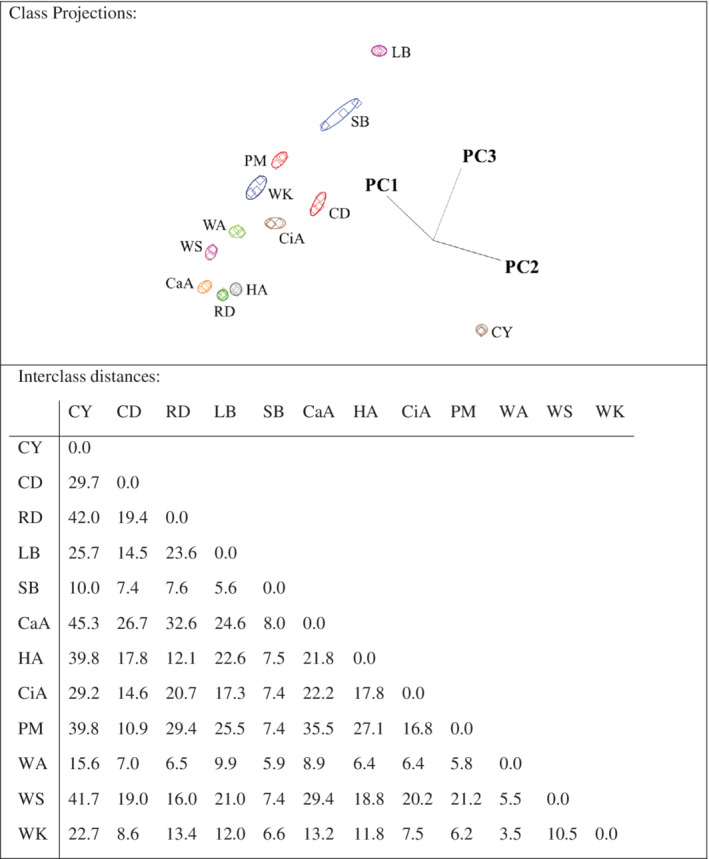
Soft independent modeling of class analogy (SIMCA) 3D projection plots of data collected by SIFT‐MS for Turkish honeys (methanol and ethanol excluded). Boundaries marked around the honey clusters represent a 95% confidence interval. Interclass distances between 12 honeys are based on the SIMCA class projections.

Multivariate statistical analyses allow the determination of botanical and geographical discrimination between honey samples. Interclass distances (ICDs) greater than 3 indicate that samples were significantly different.^32^ Better separation of honeys is achieved with higher interclass distances between two honeys. All of the ICD values of measured honeys were greater than 3, or, in this case, greater than 8 (Fig. 1), which indicates that these honey samples can be discriminated based on their volatile composition. Ethanol and methanol showed the highest discriminating power (Table 4). Because ethanol and methanol had the highest concentration of volatiles, they may cause a decrease in discriminating power, by repressing the influence of other volatiles on the volatile profile. Multivariate statistical analysis was therefore also applied to the data set without ethanol and methanol (Fig. 2). After exclusion of these two compounds, menthol, dimethyl disulfide, phenol and dimethyl sulfide showed the highest discriminating power (Table 5). Langford *et al*.^33^ also identified dimethyl disulfide as the compound with the highest discriminating power in monofloral New Zealand honeys.

**Table 4:** Discriminating power of volatile compounds of Turkish honeys

	Compounds	DP (10^2^)		Compounds (continued)	DP (10^2^)		Compounds (continued)	DP (10^2^)
1	methanol	72	19	hexanal	0.4	37	2‐heptanol	0.2
2	ethanol	21	20	acetone	0.4	38	propyl anisol	0.2
3	acetoin	16	21	isoamyl alcohol	0.4	39	2‐phenylethanol	0.2
4	ethyl acetate	16	22	vvenzyl alcohol	0.4	40	2‐aminoacetophenone	0.2
5	isobutanoic acid	16	23	1,3‐butanediol	0.3	41	heptane	0.2
6	2‐methyl‐2‐butanol	15	24	furfural	0.3	42	*cis*‐6‐nonen‐1‐ol	0.2
7	butanoic acid	15	25	2‐butanol	0.3	43	dimethyl trisulfide	0.1
8	2,3‐butanedione	14	26	menthol	0.3	44	nonanal	0.1
9	3‐methylbutanal	14	27	urfuryl alcohol	0.3	45	1‐octen‐3‐ol	0.1
10	dodecane	10	28	Isobutyl alcohol	0.3	46	coumarin	0.1
11	dimethyl sulfide	0.9	29	1‐hexanol	0.3	47	*p*‐cresol	0.1
12	acetic acid	0.8	30	benzaldehyde	0.3	48	*p*‐menth‐1‐en‐9‐al	0.1
13	2‐cyclopenten‐1,4‐dione	0.7	31	hexane	0.2	49	hotrienol	0.1
14	(*E*)‐2‐methyl‐2‐butenal	0.7	32	maltol	0.2	50	hydroxymethylfurfural	0.1
15	toluene	0.5	33	(*E*)‐2‐hexenal	0.2	51	lilac alcohol	0.1
16	phenol	0.5	34	nonane	0.2	52	octanal	0.1
17	dimethyl disulfide	0.5	35	propanoic acid	0.2			
18	(*Z*)‐3‐hexen‐1‐ol	0.5	36	lemonol	0.2			

**Table 5:** Discriminating power of volatile compounds of Turkish honeys (methanol and ethanol excluded)

	Compounds	DP (10^2^)		Compounds (continued)	DP (10^2^)		Compounds (continued)	DP (10^2^)
1	menthol	42	19	propanoic acid	2.1	37	furfural	1.3
2	dimethyl disulfide	11	20	*p*‐cresol	2.0	38	furfuryl alcohol	1.2
3	phenol	11	21	2‐methyl‐2‐butanol	1.9	39	lemonol	1.2
4	dimethyl sulfide	10	22	butanoic acid	1.9	40	1,3‐butanediol	1.2
5	benzaldehyde	9.0	23	2‐aminoacetophenone	1.9	41	heptane	1.1
6	*p*‐menth‐1‐en‐9‐al	8.2	24	hydroxymethylfurfural	1.9	42	*p*‐isopropenyl toluene	1.1
7	*p*‐mentha‐1(7),8(10)‐dien‐9‐ol	8.2	25	acetone	1.9	43	methyl anthranilate	0.8
8	hotrienol	8.2	26	isoamyl alcohol	1.9	44	2‐phenylethanol	0.8
9	toluene	6.3	27	decanal	1.8	45	(*E*)‐2‐hexenal	0.8
10	2,3‐butanedione	5.4	28	isobutyl alcohol	1.7	46	ethyl benzoate	0.7
11	1‐*p*‐menthen‐9‐ol	5.0	29	isobutanoic acid	1.7	47	damascenone	0.7
12	acetoin	4.9	30	2‐cyclopenten‐1,4‐dione	1.7	48	hexanoic acid	0.7
13	ethyl acetate	4.9	31	acetic acid	1.7	49	(*Z*)‐3‐hexen‐1‐ol	0.7
14	1‐octen‐3‐ol	4.2	32	2‐butanol	1.7	50	lilac aldehyde	0.7
15	(*E*)‐2‐methyl‐2‐butenal	4.0	33	dodecane	1.6	51	*cis*‐6‐nonen‐1‐ol	0.6
16	benzyl alcohol	3.8	34	nonanal	1.6	52	2‐hydroxyacetophenone	0.6
17	5‐methylfurfural	2.8	35	hexane	1.5			
18	dimethyl trisulfide	2.5	36	3‐methylbutanal	1.5			

When comparing different botanical sources from the same province, chestnut and rhododendron (Düzce), lavender and sage (Burdur), and carob, heather, and citrus (Antalya) showed clear differentiation (Fig. 1). The concentrations of 2‐butanol, 2‐methyl‐2‐butanol, 3‐methylbutanal, acetoin, acetone, butanoic acid, ethanol, ethyl acetate, furfuryl alcohol, isoamyl alcohol, isobutanoic acid, methanol, and propyl anisol were significantly different in chestnut and rhododendron (Düzce) (Table 3). Hexanal, hotrienol, and lilac aldehyde concentration were different in lavender and sage honey. Furfuryl alcohol is one of the characteristic compounds for chestnut honeys.^34^ Castro‐Vázquez *et al*.^8^ differentiated citrus and heather honey based on their volatile composition. Similar to our study, (*Z*)‐3‐hexen‐1‐ol, acetic acid, 2‐cyclopentene‐1‐4‐dione and butanoic acid were found to be higher in heather honey compared to citrus. These compounds were discriminating volatiles for heather and citrus. Even though it is difficult to determine the botanical source of honey accurately by many techniques,^6^ it is clearly seen that SIFT‐MS with chemometrics was effective. Agila and Barringer^18^ identified differences in the volatiles of honeys from different botanical sources (blueberry, clover, cranberry, and wildflowers) collected from the state of Indiana, USA. Langford *et al*.^33^ also applied SIFT‐MS technology to distinguish New Zealand monofloral honeys.

When the same flower source was compared with different locations, such as chestnut honeys from Yalova and Düzce, or wildflower honeys from three different provinces, varied volatile compositions were detected (Fig. 1). The composition of honey not only depends on the nectar‐providing plant species but also depends on other factors such as environmental factors, bee species, harvesting season and technology, processing, and storage.^35^ Chestnut honey collected from Yalova and Düzce regions had no statistical similarities in volatile compound concentration, except for *p*‐menth‐1‐en‐9‐al (Table 3). Chestnut honey from Yalova had a higher concentration of all compounds than chestnut honey from Düzce. The reason for this significant difference between the volatile levels was probably the geographical location. Castro‐Vázquez *et al*. 36 reported clear differentiation of chestnut honeys from different geographical origins according to their volatile composition, using multivariate statistical analysis.

The concentrations of 2,3‐butandione, 2‐butanol, 2‐cyclopenten‐1,4‐dione, acetic acid, acetoin, acetone, butanoic acid, ethyl acetate, furfural, isoamyl alcohol, isobutanoic acid, isopropyl benzene, maltol, methanol and phenylacetaldehyde were different in the three wildflower honeys from different locations. The aroma composition of wildflower honeys can be dissimilar from each other because of the variation and differences of flowers contingent upon the location.

Karabagias *et al*.^37^ investigated the geographical characterization of citrus honeys in Mediterranean countries. While ethyl acetate was determined as a key discriminating compound in citrus honeys collected from Morocco, it was not detected in honeys collected from Egypt, Greece, and Spain. Ethyl acetate was found only in Moroccan citrus honey, although ethyl octanoate and ethyl nonanoate were reported to be in higher concentration in Greek citrus honeys and ethyl nonanoate was high in Egyptian citrus honeys. Ethyl acetate may therefore be one of the compounds that can be used to geographically discriminate between Mediterranean citrus honeys.

## CONCLUSION

SIFT‐MS is a fast and simple method to enhance the difference between Turkish honeys based on their volatile composition. The application of SIFT‐MS technique with the aid of chemometrics for floral and geographical origin determination of honeys can be very useful. The data analysis takes place in two‐dimensional matrices with a chemometric approach, which allows for a better separation of the samples.

Honeys with different botanical and geographical origin showed differences in their volatile profile based on their interclass distances. Between the honey samples, methanol, ethanol, acetoin, ethyl acetate, and isobutanoic acid had the highest discriminating power and also methanol and ethanol, and then acetic acid, were the volatiles at the highest concentration in most honeys. In general, chestnut from the Yalova region had the highest total concentration of volatiles followed by heather and chestnut from the Düzce region, and wildflower from the Ardahan region had the lowest total concentration.

The volatile composition of each honey type was affected by several factors. Future studies with a broader variety of honeys or geographical origins with different harvesting seasons may be required for a better understanding of the honey fingerprint.
